# Life events and chronic physical conditions among left-behind farmers in rural China a cross-sectional study

**DOI:** 10.1186/s12889-015-1877-0

**Published:** 2015-07-01

**Authors:** Jing Chai, Penglai Chen, Rui Feng, Han Liang, Xingrong Shen, Guixian Tong, Jing Cheng, Kaichun Li, Shaoyu Xie, Yong Shi, Debin Wang

**Affiliations:** School of Health Services Management, Anhui Medical University, 81 Meishan Road, Hefei, 230032 China; Lu’an Center for Diseases Prevention and Control, Lu’an, 237000 China

**Keywords:** Life events, Chronic diseases, Risk ratio, Odds ratio, Index, Logistic regression

## Abstract

**Background:**

This study investigates the relationships between life events and chronic physical conditions among left behind farmers, a newly emerged weak group in vast rural China.

**Methods:**

The study collected information about life events, chronic physical conditions, blood pressure and fasting blood glucose from 4681 famers living in 18 randomly selected villages (Lu’an, Anhui, China) from early November 2013 to the end of December 2013. It compared the risk and odds ratios (RRs/ORs) among different subgroups divided according two life event indices derived by adding up un-weighted-ratings and weighted-ratings based on multivariate logistic regression coefficients respectively.

**Results:**

A total of 4040 (86.3 % eligible) farmers completed the survey. RRs between farmers with lower than the first 1/15-percentile of life event index and with higher life event index scores ranged 1.43–5.79 for chronic gastritis and 0.42–9.07 for prostatitis, 1.01–4.97 for cervicitis/vaginitis, 1.45–3.28 for cardio-cerebrovascular diseases, 1.12–1.58 for hypertension, 1.00–1.66 for diabetes, 1.07–3.35 for pre-diabetes and 5.00–55.00 for “other chronic physical conditions”.

**Conclusions:**

Life events were independently linked with most of the chronic physical conditions in a dose-effectiveness way. RRs between subgroups divided by given percentile cutoff points of life event index compiled using logistic regression models turned out to be substantially higher than that between subgroups divided by same cutoff points of life event index produced via summing up the un-weighted Likert ratings of all the events studied.

**Electronic supplementary material:**

The online version of this article (doi:10.1186/s12889-015-1877-0) contains supplementary material, which is available to authorized users.

## Background

Life events (LEs) and health has been researched for over five decades and continue to gain popularity worldwide [1–4]. Numerous studies suggest that acute and chronic stressful LEs are associated with a whole range of health problems including psychological and somatic impairments, cardiovascular diseases, gastrointestinal diseases, hypertension, diabetes, emphysema, asthma, and cancer [5–13]. A variety of pathways linking LEs to health problems have been proposed: a) stress derived from LEs plays an essential role in development, continuation and exacerbation of various moods and mental abnormalities [14, 15]; b) LEs activate the sympathetic nervous system and the hypothalamic-pituitary-adrenal axis which in turn cause comprised or dysfunctional immunity, elevated inflammation, reduced telomere length, and latent herpes virus reactivation [16, 17]; c) LEs induce health problems via behaviors of health significances, e.g., smoking, drug using, suicide, loosed protection against job and environment hazards, low compliance with curative and preventive interventions [18, 19].

In fact, the life-course of every person comprises complex time-series of LEs. Of these, some proceed others (e.g., marriage vs. divorce, employment vs. dismiss), while others (e.g., injury, property damage) happen stochastically; some are desirable (e.g., marriage, having a child, promotions), while others, undesirable (e.g., loss of beloved, property damage, dismiss); some (e.g., an examination, competition) end within a short time, while others (e.g., chronic pain, economic hardship) persist for years. All these features have health significance and evidences are merging that the impact of a LE is dependent upon its nature (e.g., positive vs. negative, acute vs. chronic), timing (e.g., early vs. late occurrence), intensity (e.g., severe vs. moderate impairment), presences and sequences of other LEs, as well as the individual’s resources to adapt to the event [20, 21]. In addition, the socio-cultural context also plays an important role in the links between LEs and health since it shapes the value and perception systems of the affected and related [22, 23]. For example, traditional culture in China especially rural areas values it very high that a woman should marry only one man for her whole life and therefore divorce may incur much greater stress on females than males [24].

The complex nature in the time-series of LEs and in the pathways from LEs to health makes it critical to select specific LE items and combine them into a single score for assessing their cumulative effects. On the one hand, it is impossible for any single study to tackle the whole time-series; instead, it has to be highly selective, focusing on only part of it, e.g., the major stressful LEs. On the other, any specific LE forms only a small part of the series and is subject to the influences of numerous other ones [25]. This may not only explain why published researches have documented only mixed or marginal effects but also hint much greater importance of the cumulative effect of all the LEs under concern. Contemporary views about LE selection stress both transferability and sociocultural tailoring [26]. And common strategies employed in generating collective scores are mainly counting the number of the LEs or summing up un-weighted Likert scale ratings [27]. Although these approaches are easy to use and understand, they suffer from limited ability in distinguishing overlaps, interactions and relative importance between LE items [27–29]. Given these, more and more authors are proposing relatively sophisticated methods, e.g., quartile regression, genetic algorithms and artificial neural networks [30, 31]. These models are more powerful in generating differentiated weights for combining LE items [32, 33]. Bearing these in mind, this study aims at exploring alternative approaches for assessing and analyzing LEs and their impacts. First, it tries to assess life-time accumulative effects of LEs rather than that of a limited period (e.g., 1–2 years before the study) as did in most previous studies. Second, it uses multivariate logistic regression coefficients as weights in combining individual LE items into a single index.

Another important feature that distinguishes the current study from contemporary ones relates to its focus on documenting relationships between life-time major LEs (including both positive and negative LEs) and chronic physical conditions (CPCs) among left behind farmers (LBFs) in rural Anhui, China. LBFs represent an emerging weak group living in vast rural China. The foundation, in 1949, of the new communist government paralleled establishment of a very strict residence registration system which had made migration from rural areas to cities extremely difficult and caused huge discrepancies between the two areas. This restriction has been gradually lifted starting from the late 1980s when the nation began the so called “reformation and openness” policy. And driven by rapid economic growth in cities and urbanization, most young and capable farmers move to cities for temporary jobs. LEs and health among LBFs merit special attention by several means. First, long-term separation from family members and lack of helps and care from youngsters may be profound LEs themselves. And LBFs may, due to the reverse selection, be relatively older, less capable and thus disadvantageous in copying with LEs. Second, LBFs may differ substantially from “total” rural residents that comprise both those on move to cities and those being left-behind. This may have made the existing findings about health and its influencing factors in rural China obsolete since these findings were based primarily on studies of all rural residents. Third, massive movement of farmers to cities also means huge changes in healthcare needs and demands in rural areas and thus a clear need for re-orientating the existing rural health services from “all farmers” toward LBFs. Investigating CPCs among LBFs and their relationships with LEs may not only shed new lights for understanding the effects of LEs but also call for attention for LBFs and inform service reformation in vast rural China.

## Methods

### Sampling and recruitment

This study was an integral part of an ongoing larger project aimed at developing a comprehensive instrument for assessing the risks of CPCs and identifying LBFs at elevated risk of CPCs so as to deliver targeted risk management. Sampling of site villages proceeded through: a) geographically dividing all the counties within Lu’an, one of the largest prefectures in Anhui province, China, into the north, center and south regions; b) randomly selecting 1 county from each of the regions; c) randomly selecting 1 townships from each of the counties selected; d) randomly selecting 6 villages from each of the townships selected. The research group from Anhui Medical University performed the randomization from the rosters of names of the counties, townships and villages within the selected areas provided by the local centers for disease control and prevention.

Criteria for subject inclusion were men and women who: a) had registered rural residence and were actually living in the sampled villages when this survey was conducted; b) aged 40 to 70 years; c) were willing to participate and able to answer the survey questions. The selection of the 40–70 age range was based upon: a) LBFs of 40+ was viewed as the priority group for the planned intervention since age-specific prevalence rates of most of the CPCs under concern start to increase rapidly from age 40; b) LBFs aged over 70 had already been covered by China Chronic Diseases Management Project and therefore no-longer need the intervention. Recruitment and survey of eligible LBFs started from early November 2013 and ended by the end of December 2013.

### Variables and instruments

The study comprised 5 categories of data, i.e., demographics, LEs, CPCs, and bio-physics. The LE component included 20 items (Table 1 and Additional file 1) soliciting life-time experiences of 20 events and their effects. It incorporated items from common instruments in China tailored, via qualitative interviews and pilot tests, to the local socio-cultural contexts of rural Anhui, a developing province in the middle of China [34, 35]. The qualitative interviews solicited information, from the local farmers, about what were the common life events that had significant psychological effects on and happened frequently to local farmers and whether each of the items included in the tentative life event instrument was relevant to local sociocultural contexts. The pilot tests consisted of two rounds of small-scale instrument administration, analysis and revision.

**Table 1:** Descriptive statistics of life events and chronic physical conditions by gender and age (n/%)

Life events	Gender		Age			Education			Total (n = 4040)
	Male (n = 1441)	Female (n = 2599)	40 ~ (n = 1221)	50 ~ (n = 1187)	60 ~ (n = 1632)	Illiterate (n = 1953)	Primary school (n = 1398)	≥Middle school (n = 689)	
Schooling/examination failures	377 (26.2)	697 (26.8)	307 (25.1)	368 (31.0)	399 (24.4)^††^	424 (21.7)	399 (28.5)	251 (36.4)^++^	1074 (26.6)
Abandonment of favorite pursues	282 (21.9)	504 (20.4)	237 (17.1)	273 (21.9)	334 (20.5)	385 (19.7)	283 (20.2)	176 (25.5)^++^	844 (20.9)
Punishments/dismisses	254 (17.6)	504 (19.4)	243 (19.9)	252 (21.2)	263 (16.1)^††^	379 (19.4)	260 (18.6)	119 (17.3)	758 (18.8)
Promotions/awards	241 (16.7)	100 (3.8)^**^	56 (4.6)	99 (8.3)	186 (11.4)^††^	98 (5.0)	119 (8.5)	124 (18.0)^++^	341 (8.4)
Admirable achievements	413 (28.7)	701 (27.0)	319 (26.1)	354 (29.8)	441 (27.0)	488 (25.0)	405 (29.0)	221 (32.1)^++^	1114 (27.6)
Forced/disliked endeavors	217 (15.1)	350 (13.5)	187 (15.3)	175 (14.7)	205 (12.6)	248 (12.7)	208 (14.9)	111 (16.1)^+^	567 (14.0)
Major accidents/mistakes	357 (24.8)	467 (18.0)^**^	250 (20.5)	255 (21.5)	319 (19.5)	367 (18.8)	289 (20.7)	168 (24.4)^+^	824 (20.4)
Natural disasters	631 (43.8)	989 (38.1)^**^	426 (34.9)	473 (39.8)	721 (44.2)^††^	762 (39.0)	549 (39.3)	309 (44.8)^+^	1620 (40.1)
Misunderstandings/blames	342 (23.7)	650 (25.0)	300 (24.6)	317 (26.7)	375 (23.0)	446 (22.8)	349 (25.0)	197 (28.6)^+^	992 (24.6)
Law suits due to self	104 (7.2)	58 (2.2)^**^	55 (4.5)	52 (4.4)	55 (3.4)	49 (2.5)	71 (5.1)	42 (6.1)^++^	162 (4.0)
Law suits due to relatives	33 (2.3)	96 (3.7)^*^	35 (2.9)	56 (4.7)	38 (2.3)^††^	70 (3.6)	37 (2.6)	22 (3.2)	129 (3.2)
Long-term enmities with others	189 (13.1)	321 (12.4)	93 (7.6)	184 (15.5)	233 (14.3)^††^	261 (13.4)	170 (12.2)	79 (11.5)	510 (12.6)
Marital/love breakups/conflicts	118 (8.2)	128 (4.9)^**^	112 (9.2)	68 (5.7)	66 (4.0)^††^	84 (4.3)	97 (6.9)	65 (9.4)^++^	246 (6.1)
Major injuries/diseases of relatives	528 (36.6)	1090 (41.9)^**^	473 (38.7)	430 (36.2)	715 (43.8)^††^	787 (40.3)	547 (39.1)	284 (41.2)	1618 (40.0)
Loss of relatives	1303 (90.4)	2287 (88.0)^*^	941 (77.1)	1088 (91.7)	1561 (95.6)^††^	1769 (90.6)	1224 (87.6)	597 (86.6)^++^	3590 (88.9)
Frequent parental conflicts	128 (8.9)	270 (10.4)	141 (11.5)	124 (10.4)	133 (8.1)^††^	179 (9.2)	149 (10.7)	70 (10.2)	398 (9.9)
Over worries about children	506 (35.1)	1129 (43.4)^**^	320 (26.2)	500 (42.1)	815 (49.9)^††^	912 (46.7)	510 (36.5)	213 (30.9)^++^	1635 (40.5)
Financial hardship	610 (42.3)	1195 (46.0)^*^	349 (28.6)	542 (45.7)	914 (56.0)^††^	1024 (52.4)	562 (40.2)	219 (31.8)^++^	1805 (44.7)
Stressful tasks prevailed life	174 (12.1)	603 (23.2)^**^	156 (12.8)	237 (20.0)	384 (23.5)^††^	473 (24.2)	232 (16.6)	72 (10.4)^++^	777 (19.2)
Other mis-happenings	87 (6.0)	189 (7.3)	70 (5.7)	88 (7.4)	118 (7.2)	130 (6.7)	103 (7.4)	43 (6.2)	276 (6.8)
Regression-model-based LEI (x ± SD)	0.95 ± 0.45	0.93 ± 0.45	0.79 ± 0.43	0.97 ± 0.46	1.02 ± 0.44^††^	0.94 ± 0.44	0.92 ± 0.47	0.95 ± 0.46	0.93 ± 0.45
Chronic physical conditions									
Hypertension	704 (48.9)	1043 (40.1) ^**^	318 (26.0)	531 (44.7)	898 (55.0)^††^	866 (44.3)	801 (57.3)	284 (41.2)	1747 (43.2)
Diabetes	130 (9.0)	195 (7.5)	81 (6.6)	97 (8.2)	147 (9.0)	158 (8.1)	98 (7.0)	69 (10.0)	325 (8.0)
Pre-diabetes	393 (31.6)	705 (30.6)	305 (29.0)	335 (32.0)	458 (31.6)	1111 (56.9)	706 (50.5)	338 (49.1)^++^	1098 (31.0)
Chronic gastritis	255 (17.7)	521 (20.0)	216 (17.7)	228 (19.2)	332 (20.3)	403 (20.6)	246 (17.6)	127 (18.4)	776 (19.2)
Prostatitis	131 (9.1)	NA	21 (6.0)	29 (7.1)	81 (11.9) ^††^	30 (1.5)	59 (4.2)	43 (6.2)^++^	131 (9.1)
Cervicitis/vaginitis	NA	456 (17.5)	199 (12.9)	134 (17.2)	123 (13.0) ^††^	247 (12.6)	148 (10.6)	61 (8.9)^+^	456 (17.5)
Cardio-cerebrovascular diseases	157 (10.9)	222 (8.5)^*^	64 (5.2)	95 (8.0)	220 (13.5) ^††^	178 (9.1)	134 (9.6)	67 (9.7)	379 (9.4)
Other CPCs	119 (8.3)	304 (11.7)^**^	122 (10.0)	144 (12.1)	157 (9.6)	207 (10.6)	159 (11.4)	57 (8.3)	423 (10.5)
Free from CPCs	362 (25.1)	609 (23.4)	393 (32.2)	285 (24.0)	293 (18.0) ^††^	450 (23.0)	338 (24.2)	183 (26.6)	971 (24.0)

“†” and “††” denote *p* < 0.05 and *p* < 0.01 respectively for the power test of null difference between age groups; “*” and “**”, *p* < 0.05 and *p* < 0.01, for the power test of null difference between gender groups; “^+^” and “^++^”, *p* < 0.05 and *p* < 0.01, for the power test of null difference between education groups; hypertension denotes systolic/diastolic blood pressure ≥140/90 mmHg; diabetes denotes fasting capillary glucose ≥7.0 mmol/L; pre-diabetes denotes fasting capillary glucose =[6.1, 6.9] mmol/L

Each item with our resultant LE instrument consisted of a “judging” question and a “rating” question. Taking the example of “loss of relatives”, it started with “Have you ever experienced loss of relatives like parent, spouse and children? 1) Yes; 2) No” and then followed, if the response to this “judging” question were “Yes”, by a “rating” question worded as “To what extent has the experience affected you? 1) A little; 2) Slightly; 3) Moderately; 4) Severely”.

The CPC component contained structured questions enquiring about common CPCs diagnosed or re-diagnosed/confirmed by health service providers during the past year. These common CPCs included hypertension, diabetes, chronic gastritis, prostatitis, cervicitis, vaginitis, cardio-cerebrovascular diseases, cancer and/or tumor, and others. The demographic component solicited data about age, gender and education level etc. In addition, the study also measured systolic and diastolic blood pressure (SBP/DBP) and fasting capillary glucose (FCG).

### Data collection and quality control

Data about the LEs, CPCs and demographics were collected by trained graduate students from Anhui Medical University using computerized instruments; while SBP/DBP and FCG, by trained researchers according to relevant standard procedures [36, 37]. All the interviews and measurements took place at the village clinics. Measures taken to ensure data quality included: a) training and examination of field data collectors; b) use of computerized logic checks; c) daily checks, by quality supervisors, of all the questionnaires completed during the day; d) retest of 5 % randomly selected subjects; e) feedback of errors found via the daily checks and retests; f) elimination of disqualified field data collectors.

### Value assignment and index calculation

The study used two types (one based on Likert scale sum and the other, on regression coefficients) of life event index (LEI) for quantifying total magnitude of all LEs. The Likert-scale-sum LEI followed previous studies and used the formula *LEI* = ∑_*i* = 1_^20^*x*_*i*_ [27]; while the regression-coefficients-weighted *LEI* = ∑_*i* = 1_^20^*w*_*i*_*x*_*i*_. Here, *i = i*^*th*^ item of the life events studied, *x*_*i*_ = the Likert scale rating of the *i*^*th*^ item, *W*_*i*_ = the weight of the *i*^*th*^ item generated from logistic regression modeling. The reason why we used multiple instead of binary models in calculating the weights of life events was because we thought that there may be confounding effects between specific LE and age, gender, education and other LEs. Multiple variable regression models are capable of excluding these confounding effects but not binary ones.

The modeling used “any CPC” as the dependent variable (valued as “0” if no chronic physical conditions were reported and “1” if at least one CPC was reported) and x_i_ (the Likert rating of the *i*^*th*^ item of LEs), age and gender as the covariates. The Likert scale included 5 point values, i.e., “0” if the response to the “judging” question mentioned above were “No”, “1” if the response to the “judging” question were “Yes”, and “2”, “3”, “4” and “5” if the response to the “rating” question were “A little”, “Slightly”, “Moderately” and “Severely” respectively (Additional file 1). Taking the example of the 15th item (×_15_) included in the LE instrument, it consisted of two questions and reads “Q29: Have you ever experienced loss of relatives like parent, spouse and children? 1) Yes; 2) No (Skip to Q31)” and “Q30: To what extent has the experience affected you? 1) A little; 2) Slightly; 3) Moderately; 4) Severely”. If a respondent gives “Yes” answer to Q29 and “Moderately” to Q30, then he/she adds 4 to his/her “Liker-sum LEI” or 4 × W_15_ to his/her “regression-coefficients-weighted LEI”. The resultant coefficients (or *W*_*i*_) ranged from −0.39 to 0.39 and all the coefficients greater than 0.27 and less than −0.26 were statistically significant.

### Data process and analysis

Data analysis used SPSS 16.0 (SPSS Inc., Chicago, IL, USA) and Review Manager 5.2 (Cochrane Review Manager; Cochrane Collaboration, Oxford, UK) and comprised five steps: a) descriptive summaries intended to examine distributions and patterns of the variables under concern and check for normality of the distributions; b) transformations, if necessary, to induce approximate normality; c) analysis, using two-sided test of null hypothesis, of the power of differences in occurrence of CPCs between different LE groups and relations between LEI and different CPCs; d) evaluation, via correlation analysis, of the relations between each LE item and, via binary logistic regression analysis, of the relationships between each CPC and the 20 LE items; e) estimation, using the Mantel-Haenszel method and random effects analysis model of Review Manager 5.2, of pooled risk ratios of CPCs between groups with different LEI levels.

### Research Ethics and Informed Consent

The study protocol had been reviewed and approved by the Biomedical Ethics Committee of Anhui Medical University. Participation of farmers and village doctors were all voluntary. And written informed consent was sought from all participants.

## Results

### LBFs surveyed and LEs among them

As shown in Table 1, a total of 4040 LBFs between 40 and 70 years old completed the survey. Female LBFs accounted for 64.8 %. The frequency of the LEs studied ranged from 3.2 % to 88.9 % with loss of relatives being the highest followed by financial hardship and over worries about children; while involvement in law suit, marital/love breakups or conflict and “other mis-happenings”, occurred the least. Statistically significant differences existed in 11–13 out of the 20 LE items between males and females, different age and education groups respectively.

The CronBach α of the 20-item LE instrument was estimated as 0.80. The LEI was 0.93 (95 % CI = [0.48, 1.38]) on average with an increasing trend among the age groups. Yet gender and education differences in LEI were not statistically significant. The Pearson correlation coefficients ranged from −0.037 to 0.291 between each of the LE items (Additional file 2) and from −0.008 to 0.584 between LEI and specific LE items.

### CPC prevalence by LBF groups

Table 1 also shows the prevalence rates of the common CPCs surveyed. Hypertension (SBP/DBP ≥ 140/90 mmHg) turned out to be the most common CPC (43 %), followed by pre-diabetes (FCG ≥6.1 mmol/L and ≤6.9 mmol/L) (31.0 %) and chronic gastritis (19.2 %). Compared with female LBFs, males showed significantly higher prevalence in hypertension (48.9 vs. 40.1) and cardio-cerebrovascular diseases but lower prevalence in “other CPCs” (8.3 % vs. 11.7 %). With regard to age group differences, the prevalence of hypertension, prostatitis and cardio-cerebrovascular diseases presented an increasing trend; while the proportion of LBFs who had reported no CPCs, a decreasing trend. Cervicitis and vaginitis as a whole did not show clear age trend though there existed statistically significant differences between the age groups. The prevalence of pre-diabetes among illiterate LBFs was significantly higher than other education groups. Males with higher education tended to have higher prevalence of prostatitis; while females with higher education, lower prevalence of cervicitis/vaginitis.

### Relationships between CPCs and LEs

Table 2 provides the results of multivariate logistic analysis between specific LEs and common CPCs. Controlled for age, gender and education, the ORs ranged from 0.616 (95 % CI = 0.324–1.172) to 2.245 (95 % CI = 1.483–3.398) and the majority of them were tested statistically non-significant. Looking at specific LEs, “stressful-task-prevailed life” was associated with 5 of the CPCs; followed by “schooling/examination failures”, “punishments/dismisses” and “promotions/ awards”. While “abandonment of favorite pursues”, “forced/disliked endeavors”, “law suits due to self” and “law suits due to relatives” did not show significant relations with any of the CPCs. Turning to specific CPCs, all of them had statistically significant links with 2 to 8 of the 20 LEs; yet none of them had links with all of the LEs. Other CPCs was associated with the largest number of LEs (N = 8), followed by pre-diabetes (N = 7), chronic gastritis (N = 6) and free from CPCs (N = 6).

**Table 2:** Multivariate logistic regression statistics of the relationships between life events and common chronic physical conditions

	Chronic gastritis	Prostatitis	Cervicitis/ vaginitis	Cardio/cerebro-vascular dis.	Hypertension	Diabetes	Pre-diabetes	Other CPC	Free from CPCs
Gender	1.142	NA	NA	0.847	0.828	0.806	1.113	1.464	0.794
					(0.714, 0.960)	(0.623, 1.042)	(0.962, 1.288)	(1.135, 1.889)	(0.671, 0.939)
				(0.663,1.081)					
	(0.946,1.378)								
Age	0.997	1.052	0.955	1.051	1.059	1.011	1.001	0.985	0.970
	(0.987, 1.008)	(1.025, 1.079)	(0.942, 0.968)	(1.036, 1.066)	(1.050, 1.068)	(0.996, 1.026)	(0.993, 1.009)	(0.972, 0.999)	(0.961, 0.979)
Education	0.865	1.868	0.955	1.391	1.079	0.976	0.761	0.953	0.921
	(0.724, 1.032)	(1.181,2.955)	(0.762, 1.196)	(1.096, 1.766)	(0.934, 1.246)	(0.758, 1.255)	(0.661, 0.876)	(0.757, 1.200)	(0.782, 1.085)
Schooling/examination failures	1.170	1.094	1.107	0.800	1.403	0.985	1.686	2.192	0.706
	(0.974, 1.406)	(0.715,1.671)	(0.872, 1.405)	(0.615, 1.039)	(1.202, 1.638)	(0.748, 1.298)	(1.444, 1.969)	(1.756, 2.734)	(0.585, 0.853)
Abandonment of favorite pursues	1.071	1.096	1.143	1.153	0.859	0.947	1.176	0.886	0.989
	(0.878, 1.306)	(0.701,1.712)	(0.881, 1.484)	(0.877, 1.514)	(0.724, 1.019)	(0.698, 1.283)	(0.992, 1.394)	(0.685, 1.147)	(0.807, 1.212)
Punishments/dismisses	1.310	1.016	1.206	1.089	0.792	0.710	1.232	1.129	1.028
	(1.074,1.597)	(0.624,1.654)	(0.935, 1.555)	(0.822, 1.443)	(0.666, 0.943)	(0.510, 0.987)	(1.038, 1.464)	(0.874, 1.457)	(0.840, 1.257)
Promotions/awards	1.602	0.882	1.472	0.976	1.013	0.888	1.413	1.663	0.672
	(1.212,2.117)	(0.544,1.431)	(0.898, 2.413)	(0.662, 1.438)	(0.792, 1.295)	(0.573, 1.378)	(1.101, 1.812)	(1.168, 2.368)	(0.487, 0.926)
Admirable achievements	1.255	2.245	1.262	0.862	1.106	0.956	1.283	1.161	0.900
	(1.043, 1.509)	(1.483,3.398)	(0.994, 1.602)	(0.662, 1.122)	(0.946, 1.294)	(0.723, 1.264)	(1.099, 1.499)	(0.917, 1.471)	(0.748, 1.084)
Forced/disliked endeavors	1.043	0.847	1.004	0.740	1.041	0.953	1.094	0.910	0.938
	(0.827, 1.315)	(0.495,1.449)	(0.741,1.360)	(0.520, 1.052)	(0.851, 1.272)	(0.666, 1.364)	(0.894, 1.337)	(0.676, 1.226)	(0.736, 1.196)
Major accidents/mistakes	1.202	1.235	1.121	1.011	1.040	0.908	1.001	1.136	0.782
	(0.989, 1.461)	(0.808,1.888)	(0.861, 1.460)	(0.771, 1.325)	(0.882, 1.227)	(0.677, 1.219)	(0.849, 1.179)	(0.883, 1.462)	(0.641, 0.955)
Natural disasters	1.090	1.428	1.030	1.206	1.143	0.904	1.165	0.913	0.925
	(0.923, 1.288)	(0.973,2.097)	(0.828, 1.282)	(0.965, 1.506)	(0.998, 1.309)	(0.710, 1.151)	(1.019, 1.331)	(0.734, 1.137)	(0.790, 1.082)
Misunderstandings/blames	0.945	0.890	1.181	1.192	0.948	0.994	1.066	1.285	0.920
	(0.776, 1.151)	(0.564,1.403)	(0.923, 1.513)	(0.915, 1.555)	(0.804, 1.118)	(0.744, 1.328)	(0.905, 1.255)	(1.010, 1.634)	(0.757, 1.120)
Law suits due to self	1.221	1.114	0.686	0.616	1.183	1.151	0.820	1.054	1.094
	(0.826, 1.806)	(0.554,2.240)	(0.334, 1.410)	(0.324, 1.172)	(0.843, 1.659)	(0.664, 1.998)	(0.586, 1.147)	(0.630, 1.761)	(0.740, 1.617)
Law suits due to relatives	0.622	0.674	1.063	0.719	0.753	1.101	1.007	0.974	1.484
	(0.380,1.018)	(0.193,2.356)	(0.633, 1.784)	(0.356, 1.453)	(0.515, 1.102)	(0.594, 2.044)	(0.695, 1.457)	(0.563, 1.682)	(0.986, 2.235)
Long term enmities with others	1.269	1.326	1.117	1.053	1.031	1.380	1.085	1.347	0.771
	(1.003, 1.605)	(0.797,2.206)	(0.813, 1.534)	(0.762, 1.455)	(0.839, 1.267)	(0.987, 1.930)	(0.881, 1.337)	(1.009, 1.800)	(0.591, 1.006)
Marital/love breakups/conflicts	0.953	2.203	1.019	0.853	1.082	1.213	1.146	1.852	0.872
	(0.684, 1.327)	(1.228,3.950)	(0.654, 1.587)	(0.517, 1.405)	(0.819, 1.429)	(0.772, 1.906)	(0.867, 1.513)	(1.299, 2.641)	(0.626, 1.214)
Major injuries/diseases of relatives	0.913	1.123	1.267	1.146	1.075	1.266	0.978	1.065	0.929
	(0.773, 1.078)	(0.761,1.656)	(1.025, 1.567)	(0.918, 1.430)	(0.939, 1.230)	(1.002, 1.601)	(0.857, 1.117)	(0.860, 1.318)	(0.795, 1.086)
Loss of relatives	1.198	1.110	1.215	1.550	1.251	0.902	1.142	1.811	0.679
	(0.897, 1.599)	(0.507,2.433)	(0.869, 1.700)	(0.961, 2.499)	(0.996, 1.571)	(0.619, 1.314)	(0.925, 1.410)	(1.179, 2.783)	(0.544, 0.846)
Frequent parental conflicts	1.116	0.652	1.259	0.812	1.154	1.115	0.796	1.176	0.892
	(0.862, 1.444)	(0.314,1.356)	(0.920, 1.721)	(0.549, 1.202)	(0.925, 1.441)	(0.765, 1.626)	(0.639, 0.991)	(0.860, 1.609)	(0.683, 1.165)
Over worries about children	1.250	1.252	1.132	1.271	1.080	0.844	1.118	1.728	0.836
	(1.051, 1.487)	(0.844,1.857)	(0.899, 1.426)	(1.009, 1.600)	(0.937, 1.245)	(0.654, 1.087)	(0.971, 1.286)	(1.377, 2.169)	(0.708, 0.988)
Financial hardship	1.013	0.860	0.860	0.919	0.923	0.931	1.145	0.968	0.969
	(0.851, 1.206)	(0.579,1.277)	(0.682, 1.085)	(0.729, 1.158)	(0.801, 1.063)	(0.726, 1.194)	(0.997, 1.316)	(0.770, 1.215)	(0.823, 1.141)
Stressful tasks prevailed life	1.684	1.130	1.633	1.482	0.975	1.155	1.476	1.258	0.758
	(1.381, 2.054)	(0.666,1.915)	(1.266, 2.106)	(1.132, 1.940)	(0.817, 1.163)	(0.851, 1.567)	(1.234, 1.765)	(0.975, 1.623)	(0.606, 0.948)
Other mis-happenings	1.092	1.047	1.268	1.467	0.999	1.636	0.989	1.469	0.759
	(0.809,1.473)	(0.517,2.118)	(0.877, 1.834)	(1.005, 2.142)	(0.770, 1.296)	(1.109, 2.414)	(0.763, 1.282)	(1.042, 2.070)	(0.542, 1.062)
Constant	0.120	0.002	1.259	0.004	0.029	0.076	0.543	0.034	5.633

Hypertension denotes systolic/diastolic blood pressure ≥ 140/90 mmHg; diabetes denotes fasting capillary glucose ≥7.0 mmol/L; pre-diabetes denotes fasting capillary glucose = [6.1, 6.9] mmol/L

### Relationships between CPCs and LEI

**Fig. 1:**
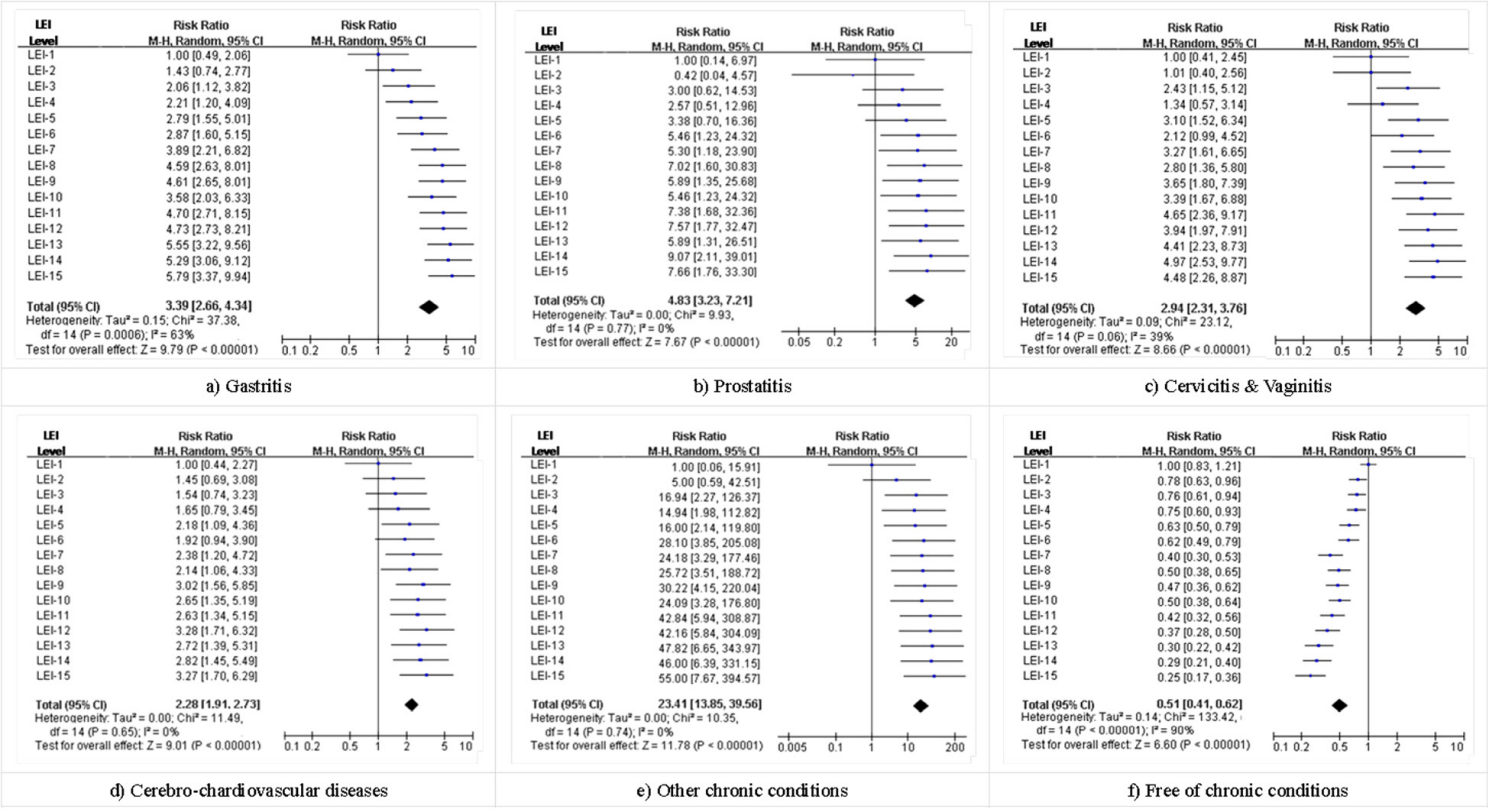
Forest plots of risk ratios of chronic physical conditions between different farmers groups. LEI=Life event index produced using logistic regression coefficients; LEI-1 through LEI-15 stands for the first through the fifteenth 1/15-percentile LEI respectively; all the RRs were estimated using farmers with LEI-1 as base group and that with LEI-2 through LEI-15 as comparison group

Figure 1 depicts selective (rather than all, due to space limit) forest plots of RRs between different farmer groups. By contrasting the RRs of a given CPC between the LBFs with reference LEI (LEI-1) and that with LEI-2 through LEI-15, these figures reveal apparent “dose-effectiveness” relationships, i.e., the higher the LEI of the LBFs, the greater the chance they were suffering from the CPC. And this applied to all the CPCs included in this study. Yet this relationship manifested substantial differences across CPCs. The highest comparative (LEI-2 through LEI-15 vs. LEI-1) RRs of specific CPCs ranged from 1.58 (95 % CI = [1.29, 1.94]) for hypertension to 55.00 (95 % CI = [7.67, 394.57]) for “other CPCs”.

Table 3 provides the RRs and 95 % confidence intervals of different CPCs between the LBFs grouped by the two types of LEI described in the methodology section. Almost all the RRs between groups classified using the logistic-regression-based LEI were greater than that between groups classified using the Likert-scale-sum LEI.

**Table 3:** Risk ratios (RRs) of chronic physical conditions (CPCs) between different farmer groups

CPCs and type of LEIs	LEI-1	LEI-2	LEI-3	LEI-4	LEI-5	LEI-6	LEI-7	LEI-8	LEI-9	LEI-10	LEI-11	LEI-12	LEI-13	LEI-14	LEI-15
Likert Scale Sum-based LEI															
- Chronic gastritis	1.00	1.37	1.93	2.07	3.37	2.50	2.67	2.24	3.36	2.99	3.18	3.79	3.37	3.73	4.64
	(0.56, 1.79)	(0.80, 2.34)	(1.18, 3.15)	(1.28, 3.34)	(2.09, 5.44)	(1.57, 3.98)	(1.62, 4.43)	(1.33, 3.78)	(2.13, 5.30)	(1.87, 4.76)	(1.97, 5.13)	(2.37, 6.05)	(2.08, 5.48)	(2.35, 5.90)	(2.95, 7.29)
- Prostatitis	1.00	1.51	0.83	2.78	2.12	2.62	2.27	2.02	2.20	2.93	2.50	1.86	4.03	4.14	2.27
	(0.30, 3.37)	(0.51, 4.50)	(0.25, 2.81)	(1.06, 7.32)	(0.70, 6.44)	(0.99, 6.94)	(0.72, 7.15)	(0.64, 6.37)	(0.78, 6.24)	(1.05, 8.12)	(0.87, 7.21)	(0.56, 6.20)	(1.35, 12.03)	(1.54, 11.15)	(0.72, 7.15)
- Cervicitis/vaginitis	1.00	2.05	2.01	2.24	2.04	1.96	2.43	3.58	3.04	2.94	4.23	3.46	3.70	4.13	4.05
	(0.45, 2.25)	(1.02, 4.09)	(1.02, 3.96)	(1.16, 4.33)	(0.98, 4.24)	(1.01, 3.79)	(1.21, 4.89)	(1.86, 6.89)	(1.61, 5.75)	(1.55, 5.57)	(2.24, 7.99)	(1.81, 6.63)	(1.95, 7.05)	(2.22, 7.69)	(2.16, 7.61)
- Cardio/cerebro-vascular diseases	1.00	0.72	1.06	1.24	1.05	1.11	1.38	1.54	1.27	1.56	1.67	1.46	1.43	1.44	1.44
	(0.57, 1.77)	(0.39, 1.34)	(0.62, 1.82)	(0.74, 2.08)	(0.57, 1.95)	(0.66, 1.86)	(0.77, 2.46)	(0.88, 2.69)	(0.75, 2.14)	(0.94, 2.59)	(0.98, 2.83)	(0.84, 2.55)	(0.80, 2.55)	(0.85, 2.46)	(0.83, 2.49)
- Hypertension	1.00	1.06	0.91	0.95	1.06	1.05	0.96	0.99	1.26	1.20	1.10	1.14	1.18	1.15	1.19
	(0.82, 1.22)	(0.88, 1.29)	(0.74, 1.10)	(0.79, 1.15)	(0.85, 1.30)	(0.88, 1.26)	(0.76, 1.20)	(0.79, 1.23)	(1.06, 1.51)	(1.00, 1.44)	(0.90, 1.35)	(0.93, 1.39)	(0.96, 1.45)	(0.95, 1.40)	(0.98, 1.45)
- Diabetes	1.00	1.51	1.12	1.04	1.53	1.48	1.70	1.30	2.02	1.44	1.77	1.81	1.39	1.07	1.08
	(0.52, 1.92)	(0.84, 2.73)	(0.60, 2.06)	(0.56, 1.92)	(0.81, 2.90)	(0.84, 2.60)	(0.90, 3.18)	(0.66, 2.54)	(1.16, 3.51)	(0.80, 2.61)	(0.97, 3.24)	(0.98, 3.32)	(0.71, 2.71)	(0.55, 2.07)	(0.55, 2.15)
- Pre-diabetes	1.00	0.99	0.97	1.30	1.43	1.44	1.46	1.29	1.62	1.47	1.67	1.68	1.56	1.86	1.79
	(0.81, 1.23)	(0.81, 1.22)	(0.79, 1.19)	(1.09, 1.55)	(1.18, 1.74)	(1.21, 1.71)	(1.21, 1.78)	(1.05, 1.58)	(1.37, 1.92)	(1.23, 1.76)	(1.40, 2.00)	(1.41, 2.01)	(1.29, 1.88)	(1.58, 2.20)	(1.50, 2.12)
- Other CPCs	1.00	2.84	0.83	0.82	1.89	2.45	3.31	2.10	3.85	2.66	1.74	2.33	7.20	3.18	7.56
	(0.26, 3.91)	(0.94, 8.55)	(0.21, 3.27)	(0.21, 3.20)	(0.52, 6.83)	(0.81, 7.41)	(1.01, 10.88)	(0.58, 7.57)	(1.31, 11.35)	(0.83, 8.56)	(0.48, 6.29)	(0.65, 8.37)	(2.39, 21.75)	(0.99, 10.21)	(2.64, 21.67)
- Free from CPCs	1.00	0.94	1.01	1.01	0.97	0.95	0.92	0.96	0.90	0.94	0.97	0.95	0.79	0.93	0.78
	(0.95, 1.05)	(0.88, 1.00)	(0.96, 1.05)	(0.96, 1.05)	(0.91, 1.04)	(0.90, 1.01)	(0.84, 1.01)	(0.90, 1.03)	(0.83, 0.98)	(0.88, 1.01)	(0.92, 1.04)	(0.88, 1.03)	(0.66, 0.94)	(0.85, 1.01)	(0.67, 0.90)
Logistic regression-based LEI															
- Chronic gastritis	1.00	1.43	2.06	2.21	2.79	2.87	3.89	4.59	4.61	3.58	4.70	4.73	5.55	5.29	5.79
	(0.49, 2.06)	(0.74, 2.77)	(1.12, 3.82)	(1.20, 4.09)	(1.55, 5.01)	(1.60, 5.15)	(2.21, 6.82)	(2.63, 8.01)	(2.65, 8.01)	(2.03, 6.33)	(2.71, 8.15)	(2.73, 8.21)	(3.22, 9.56)	(3.06, 9.12)	(3.37, 9.94)
- Prostatitis	1.00	0.42	3.00	2.57	3.38	5.46	5.30	7.02	5.89	5.46	7.38	7.57	5.89	9.07	7.66
	(0.14, 6.97)	(0.04, 4.57)	(0.62, 14.53)	(0.51, 12.96)	(0.70, 16.36)	(1.23, 24.32)	(1.18, 23.90)	(1.60, 30.83)	(1.35, 25.68)	(1.23, 24.32)	(1.68, 32.36)	(1.77, 32.47)	(1.31, 26.51)	(2.11, 39.01)	(1.76, 33.30)
- Cervicitis/vaginitis	1.00	1.01	2.43	1.34	3.10	2.12	3.27	2.80	3.65	3.39	4.65	3.94	4.41	4.97	4.48
	(0.41, 2.45)	(0.40, 2.56)	(1.15, 5.12)	(0.57, 3.14)	(1.52, 6.34)	(0.99, 4.52)	(1.61, 6.65)	(1.36, 5.80)	(1.80, 7.39)	(1.67, 6.88)	(2.36, 9.17)	(1.97, 7.91)	(2.23, 8.73)	(2.53, 9.77)	(2.26, 8.87)
- Cardio/cerebro-vascular diseases	1.00	1.45	1.54	1.65	2.18	1.92	2.38	2.14	3.02	2.65	2.63	3.28	2.72	2.82	3.27
	(0.44, 2.27)	(0.69, 3.08)	(0.74, 3.23)	(0.79, 3.45)	(1.09, 4.36)	(0.94, 3.90)	(1.20, 4.72)	(1.06, 4.33)	(1.56, 5.85)	(1.35, 5.19)	(1.34, 5.15)	(1.71, 6.32)	(1.39, 5.31)	(1.45, 5.49)	(1.70, 6.29)
- Diabetes	1.00	1.00	1.41	1.26	1.29	1.18	1.01	1.07	1.66	1.12	1.23	1.54	1.64	1.53	1.24
	(0.52, 1.92)	(0.52, 1.92)	(0.77, 2.56)	(0.67, 2.34)	(0.70, 2.38)	(0.63, 2.20)	(0.53, 1.93)	(0.56, 2.05)	(0.93, 2.96)	(0.60, 2.11)	(0.66, 2.28)	(0.85, 2.76)	(0.92, 2.93)	(0.85, 2.75)	(0.67, 2.29)
- Pre-diabetes	1.00	1.07	1.48	2.06	1.93	1.97	2.13	2.18	2.43	2.83	2.84	3.35	3.06	2.96	3.33
	(0.60, 1.66)	(0.65, 1.76)	(0.93, 2.33)	(1.33, 3.17)	(1.25, 2.97)	(1.28, 3.03)	(1.39, 3.25)	(1.42, 3.35)	(1.60, 3.68)	(1.88, 4.24)	(1.90, 4.26)	(2.25, 4.97)	(2.05, 4.57)	(1.98, 4.43)	(2.24, 4.95)
- Other CPCs	1.00	5.00	16.94	14.94	16.00	28.10	24.18	25.72	30.22	24.09	42.84	42.16	47.82	46.00	55.00
	(0.06, 15.91)	(0.59, 42.51)	(2.27, 126.37)	(1.98, 112.82)	(2.14, 119.80)	(3.85, 205.08)	(3.29, 177.46)	(3.51, 188.72)	(4.15, 220.04)	(3.28, 176.80)	(5.94, 308.87)	(5.84, 304.09)	(6.65, 343.97)	(6.39, 331.15)	(7.67, 394.57)
- Free from CPCs	1.00	0.78	0.76	0.75	0.63	0.62	0.40	0.50	0.47	0.50	0.42	0.37	0.30	0.29	0.25
	(0.83, 1.21)	(0.63, 0.96)	(0.61, 0.94)	(0.60, 0.93)	(0.50, 0.79)	(0.49, 0.79)	(0.30, 0.53)	(0.38, 0.65)	(0.36, 0.62)	(0.38, 0.64)	(0.32, 0.56)	(0.28, 0.50)	(0.22, 0.42)	(0.21, 0.40)	(0.17, 0.36)
- Hypertension	1.00	1.24	1.19	1.12	1.18	1.15	1.38	1.31	1.25	1.45	1.40	1.48	1.38	1.58	1.52
	(0.79, 1.27)	(0.99, 1.54)	(0.95, 1.49)	(0.88, 1.41)	(0.94, 1.48)	(0.92, 1.45)	(1.11, 1.71)	(1.05, 1.64)	(1.00, 1.56)	(1.18, 1.80)	(1.13, 1.73)	(1.20, 1.82)	(1.11, 1.71)	(1.29, 1.94)	(1.23, 1.87)

LEI-1 through LEI-15 stands for the first through fifteenth 1/15-percentile of LEI respectively; hypertension denotes systolic/diastolic blood pressure ≥ 140/90 mmHg; diabetes denotes fasting capillary glucose ≥7.0 mmol/L; pre-diabetes denotes fasting capillary glucose = [6.1, 6.9] mmol/L

Table 4 presents the main results of multivariate logistic regression analysis of the relationships between LEI and CPCs. After exclusion of potential effects of age, gender and education, the relationships remained statistically significant and the steadily growing trend in the risks from the group with the lowest LEI to that with the highest LEI discovered through the above bivariate comparative analysis was still observable for almost all of the CPCs except for diabetes. For chronic gastritis, prostatitis and “other CPCs”, the ORs between LEI-1 vs. LEI-2 through LEI-15 groups showed even greater odds ratios (ORs) than the corresponding RRs in Table 3, being 1.487 to 7.873, 1.707 to 11.670 and 5.400 to 78.505 respectively. By splitting the LBFs into two different age groups and calculating the same ORs as that shown in Table 4, almost all of the CPCs exhibited a general trend of greater ORs among the LBFs aged 40–55 years than that among those aged 56–70 years (Additional file 3).

**Table 4:** Multivariate logistic regression statistics of the relationships between life-event index and common chronic physical conditions

Farmer groups	Chronic gastritis		Prostatitis		Cervicitis/ vaginitis		Cardio/cerebro-vascular dis.		Hypertension		Diabetes		Pre-diabetes		Other CPCs		Free from CPCs	
	B	EXP (B)	B	EXP (B)	B	EXP (B)	B	EXP (B)	B	EXP (B)	B	EXP (B)	B	EXP (B)	B	EXP (B)	B	EXP (B)
Gender	0.055	1.057	NA	NA	NA	NA	−0.101	0.904	−0.198	0.820^**^	−0.149	0.862	−0.091	0.913	0.205	1.228	−0.159	0.853
Age	−0.004	0.996	0.037	1.038^**^	−0.051	0.950^**^	0.054	1.056^**^	0.058	1.060^**^	0.009	1.009	−0.001	0.999	−0.020	0.980^**^	−0.032	0.969^**^
Education	−0.066	0.936	0.272	1.313^*^	0.052	1.054	0.168	1.183^*^	0.083	1.086	0.058	1.060	−0.010	0.990	−0.068	0.935	−0.055	0.947
LEI-2	0.397	1.487	0.535	1.707	0.140	1.150	0.297	1.346	0.216	1.241	−0.026	0.974	0.018	1.018	1.686	5.400	−0.355	0.701^*^
LEI-3	0.758	2.134^*^	1.060	2.887	1.143	3.137^*^	0.314	1.369	0.105	1.110	0.348	1.417	0.491	1.635^*^	2.954	19.188^**^	−0.371	0.690^*^
LEI-4	0.830	2.292^*^	0.675	1.964	0.463	1.588	0.332	1.393	0.002	1.002	0.221	1.247	0.720	2.054^**^	2.820	16.780^**^	−0.376	0.687^*^
LEI-5	1.125	3.079^**^	1.636	5.134	1.436	4.205^**^	0.741	2.097	0.146	1.157	0.265	1.303	0.654	1.924^**^	2.863	17.518^**^	−0.660	0.517^**^
LEI-6	1.169	3.220^**^	1.475	4.369	1.009	2.742^*^	0.516	1.675	0.011	1.011	0.147	1.159	0.679	1.972^**^	3.466	32.008^**^	−0.618	0.539^**^
LEI-7	1.537	4.650^**^	2.376	10.766^*^	1.552	4.720^**^	0.731	2.078	0.387	1.473^*^	−0.012	0.989	0.666	1.947^**^	3.333	28.028^**^	−1.222	0.295^**^
LEI-8	1.748	5.742^**^	2.464	11.747^*^	1.407	4.083^**^	0.605	1.831	0.222	1.249	0.038	1.039	0.820	2.271^**^	3.413	30.369^**^	−0.900	0.406^**^
LEI-9	1.772	5.885^**^	2.184	8.883^*^	1.790	5.990^**^	0.974	2.649^**^	0.090	1.094	0.512	1.668	0.905	2.473^**^	3.599	36.551^**^	−0.977	0.376^**^
LEI-10	1.437	4.206^**^	3.121	22.669^**^	1.727	5.621^**^	0.842	2.322^*^	0.392	1.480^*^	0.029	1.029	0.989	2.689^**^	3.364	28.895^**^	−0.876	0.417^**^
LEI-11	1.771	5.877^**^	2.443	11.504^*^	2.083	8.027^**^	0.834	2.302^*^	0.334	1.396	0.194	1.214	1.003	2.726^**^	3.996	54.355^**^	−1.095	0.335^**^
LEI-12	1.777	5.915^**^	2.303	10.001^*^	1.895	6.651^**^	1.098	2.999^**^	0.450	1.568^*^	0.432	1.541	1.268	3.553^**^	4.002	54.697^**^	−1.271	0.281^**^
LEI-13	2.003	7.411^**^	3.036	20.830^**^	1.984	7.273^**^	0.843	2.323^*^	0.270	1.310	0.484	1.623	1.178	3.249^**^	4.153	63.617^**^	−1.459	0.233^**^
LEI-14	1.944	6.985^**^	2.567	13.022^*^	2.289	9.869^**^	0.843	2.323^*^	0.503	1.654^**^	0.429	1.535	1.116	3.052^**^	4.125	61.866^**^	−1.469	0.230^**^
LEI-15	2.063	7.873^**^	2.457	11.670^*^	2.233	9.324^**^	0.968	2.633^**^	0.330	1.391	0.175	1.192	1.230	3.421^**^	4.363	78.505^**^	−1.625	0.197^**^
Constant	−2.686	0.068^**^	−7.199	0.001^**^	−0.463	0.629	−6.215	0.002^**^	−3.578	0.028^**^	−3.023	0.049^*^	−1.409	0.245^**^	−1.568	0.209^**^	1.767	5.853^**^

“*” and “**” denote *p*<0.05 and *p*<0.01 respectively for the power test of null difference between age groups. LEI stands for combined life event index and LEI-1 through to LEI-15, first through to fifteenth 15 percentile of LEI respectively; hypertension denotes systolic/diastolic blood pressure ≥ 140/90 mmHg; diabetes denotes fasting capillary glucose ≥7.0 mmol/L; prediabetes denotes fasting capillary glucose = [6.1, 6.9] mmol/L

## Discussion

This study revealed apparent, independent and “dose-effectiveness” trend in the relationships between LEI and relative risks for reporting CPCs among LBFs. This is noteworthy given that most previous research findings in this regard have been inconclusive [7, 9, 10, 15]. This may due largely to the methods used in combining individual LE items into a single index (LEI) and psycho-social contexts of the subjects we had studied. Both counting the number and summing up the Likert scale ratings of LEs, as being applied in most contemporary studies, treat all individual LEs as equal. This is often inappropriate, since the impact of different LEs varies greatly [16, 38]. Weighing the LE items according to multivariate logistic regression coefficients seemed to be an effective approach in assessing collective effects of multiple items of LEs. Our analysis showed that the majority of the RRs between LBFs grouped according to regression coefficients-based LEI were greater than that grouped according to Likert scale sum. Besides, the factors causing the farmers to be left behind may also have important psychological significances. Being less capable or confident in finding jobs in cities may also mean poorer resources, ability and efficacy etc. for copying with LEs. And, as mentioned earlier, being left behind parallels long-term separation and lack of helps and care from family members, which may all be profound LEs themselves. The number (i.e., 15) of subgroups used for paired-comparisons to disclose RRs/ORs of the CPCs was a balanced consideration of two factors. On one hand, larger number of subgroups means larger potential LEI discrepancies between the baseline and the remaining groups (e.g., the first vs. the last group) and hence larger chances for finding greater mean RRs/ORs. On the other, as the number of subgroups increases, the number of LBFs falling into each subgroup decreases and thus reduces the power for identifying statistically significant differences. In addition, the general trend of greater ORs among the LBFs aged 40–55 years than that among those aged 56–70 years may suggest potential differences in the magnitude and/or mechanisms of LE effects across the stages of life course.

Another point worth noting referrers to the huge discrepancies in the RRs or ORs for different CPCs, e.g., from RR = 1.58 (1.29, 1.94) in our bivariate analysis (or OR = 1.65, in our multi regression analysis) for hypertension to RR = 55.00(7.67, 394.57) (or OR = 78.51) for “other CPCs”. Although numerous studies have already documented similar results (e.g., Quendo MA et al. reported OR = 4.83 for suicidal behavior; while Pietrzak and colleagues, OR = 1.8 for gastritis), this study provided a good opportunity for comparing LE effects on different health problems [39, 40]. Contrary to most previous studies, which generally focused on a singular health problem, this study included a set of CPCs at the same time and thus enabled generating RRs/ORs of different diseases from a same research design, LE instrument, population group etc. The RRs/ORs of some specific CPCs (e.g. prostatitis, cervicitis/vaginitis and chronic gastritis) turned out to be apparently higher than that of others (e.g. diabetes and hypertension). This may be attributed partly to differences in the paths from LEs to different CPCs as mentioned earlier and partly, differences in the prevalence rates of the CPCs that may result in different chances of random errors. One possible explanation for the only null relationship between LEI levels and RRs/ORs of diabetes may be that some of the previously diagnosed diabetics may have been taking glucose lowering medications and/or practicing lifestyle modifications that had resulted in lower FCG. This may also apply to the relatively low RRs/ORs of hypertension.

The third point worth noting concerns a subtle yet important difference between the effects of LEs assessed in this study and that in contemporary ones. Most previous studies asked occurrence of LEs within a limited period (typically 1–2 years) before a given time point (usually when the first wave field data collection was executed) and onset of certain diseases afterwards [7, 11]. Such a research design may be advantageous for probing causal relations; yet it considers only limited LEs and incomplete (mostly immediate but long-term) health effects. This study analyzed relationships between “life-time” LEs and occurrence of the CPCs in the previous year and therefore took into account accumulated effects of all the LEs on the CPCs studied. Given that chronic diseases generally develop over many years, exploring the long-term accumulative effects of LEs may be more important than the immediate effects. And inferring from the various pathways linking LEs to health problems summarized in the introduction section, there are reasons to believe that LEs can have such long-term effects. For example, schooling/ examination failures may not only have immediate health effects (within a few months after the event) but also sustained or repeated effects under certain circumstances. Schooling/examination failures in China determine whether or not an individual can enter most wanted study programs, professions or jobs and are valued high by all Chinese; and these examinations are repeated annually and are widely covered by the media each time. These may make those who had failed the same examinations recall their own failures year after year and thus cause repeated distresses or bad feelings. Schooling/examination failures may lead to higher life-time risk of other potential LEs (e.g., social discriminations, bad job performances, low self-esteem). Schooling/examination failures may also mean less life-time ability coping with potential LEs. In addition, schooling/examination failures may be linked with increased unhealthy behaviors including smoking, sex for money/shelving, low fruit and vegetable intake, under-utilization of health services etc.

The fourth point worth mentioning relates to the LE instrument used. It consisted of 20 items designed as an interviewer-administered questionnaire to suit highly illiterate LBFs. As mentioned earlier in the methods section, each of the instrument items divided into two parts, i.e. a “judging” question followed by a “rating” question (Additional file 1). This arrangement facilitated the interview process since: a) the “judging” question with the simplest responses (“Yes” or “No”) enabled rapid skipping of unnecessary “rating” questions; b) the identical “rating” questions made, after completion of the first few items, the respondent readily prepared for what to response after he/she had given an “Yes” answer. As a result, the instrument administration took only about 5 to 10 min. The standardized CronBach α (0.80) suggests that the instrument is quite reliable; while the correlations coefficients (from −0.037 to 0.291) between the 20 LEs (Additional file 2) indicate that all the items included in the instrument are relatively independent.

In addition, this study documented preliminary information about the prevalence of the CPCs and LEs among all the LBFs and different subgroups. For instances, the study found that: a) the prevalence of hypertension was apparently higher among the LBFs (43.2 %) than the national average (26.6 %) of the same age range [41]; b) about 31 % of the LBFs were tested with pre-diabetes yet had never known their glucose status before; c) “loss of relatives’, “financial hardship”, “over worries about children”, “major injuries/diseases of relatives” and “natural disasters” were most prevalent among the LBFs. Putting together, these findings and others not only call for attention to LE-related issues among LBFs, a newly emerged and thus relatively neglected weak group in vast rural China, but also inform similar studies in the future.

The current study has several strengths. First, it explored the relationships between LEs and CPCs among emerging and relatively neglected weak group. Second, it utilized a tailored instrument for assessing LEs and their effects. Third, it produced and compared two indices for evaluating the cumulative effects of LEs. Fourth, it focused on LEs happened during lifetime rather than within a limited period before a given time point.

This study also suffers from limitations. First, except for hypertension, diabetes and pre-diabetes, the remaining CPCs were all reported chronic conditions that had been diagnosed by doctors before the interview. This raises a number of concerns about biases: a) the criteria used for diagnosing the CPCs may be different across service providers; b) the recall ability and service seeking behavior may differ across LBFs. Second, the relationships between LEs and CPCs are bidirectional in nature and readers are cautioned about the difficulties in inferring cause-and-effect relations using data derived from the cross-sectional design [34, 35]. Third, the current study solicited information about life-time LEs and CPCs diagnosed within the past year. Such a research design makes it difficult to tell whether some of the LEs happened before or after the CPCs, though this difficulty applies to only a very small proportion 1.4–2.5 % (i.e., 1/40 to 1/70) of all the LEs experienced by the LBFs. Fourth, after decades of the internal migration, a highly selective process, the LBFs studied characterized lower education, poorer health and over representation of females etc. These all have implications for interpreting and generalizing the findings. Fifth, the over-representation of female LBFs may bias our findings from a comparative stand point. For example, it may be inappropriate to compare the prevalence rates of LEs and CPCs among our study population as a whole and populations with approximately equal gender compositions. Sixth, although the regression model-based weighing of individual LEs has resulted in seeming better findings than that of traditional methods, it needs to be further validated since there is little previous literature endorsing the method. Last, the study site, Lu’an, locates in the middle of China. It represents typical inland rural areas in the country. Yet the findings should be generalized with caution to costal or boarder areas of the country.

### Conclusions and implications

LEs among LBFs in rural Anhui, China were independently related to most common CPCs in a dose-effectiveness way. This relationship varied greatly across CPCs. And RRs between subgroups of LBFs divided by given percentile cutoff points of LEI compiled using logistic regression models turned out to be substantially higher than that between subgroups divided by same cutoff points of LEI produced via summing up the Likert ratings of all the events studied.

These findings have important implications for clinicians and policymakers. Clinicians, especially those in rural areas, may need to bear in mind the significance of LEs to the health of their patients and take LE history into account in preventing, diagnosing and treating CPCs. Similarly, policymakers may need to be fully aware of the radical changes in the composition of farmers, the high prevalence of CPCs among them and the roles of LEs in the epidemics, and take concrete measures in reforming rural health services and addressing LE-related health problems.

## Additional files

Additional file 1:
**Life event instrument among left-behind farmers in rural China.**
Additional file 2:
**Correlation coefficients between each of the 20 life event items.**
Additional file 3:
**Age stratified odd ratios of chronic physical conditions between left behind farmers with different life event index.**

## Abbreviations

LEs: Life events
LEI: Life event index
CPCs: Chronic physical conditions
RR: Risk ratio
OR: Odds ratio
FBG: Fasting capillary blood glucose
