# Supplementary material for: Life events and chronic physical conditions among left-behind farmers in rural China a cross-sectional study
Source: BMC Public Health. 2015 Jul 1;15:594. doi: 10.1186/s12889-015-1877-0 (PMC4487061; doi:10.1186/s12889-015-1877-0)
Supplement: Additional file 1: — Life event instrument among left-behind farmers in rural China. [file 12889_2015_1877_MOESM1_ESM.doc]

## Additional file 1: Life event instrument among left-behind farmers in rural China

Q1: Have you ever experienced schooling and major examination failures in your lifetime?

1) Yes (Q1=1 if checked or 0 if not checked)

2) No (Skip to Q3)

Q2: To what extent has the experience affected you?

1) A little (Q2=2 if checked or 0 if not checked)

2) Slightly (Q2=3 if checked or 0 if not checked)

3) Moderately (Q2=4 if checked or 0 if not checked)

4) Severely (Q2=5 if checked or 0 if not checked)

Q3: Have you ever experienced abandonment of major favorite pursues in your lifetime?

1) Yes (Q3=1 if checked or 0 if not checked)

2) No (Skip to Q5)

Q4: To what extent has the experience affected you?

1) A little (Q4=2 if checked or 0 if not checked)

2) Slightly (Q4=3 if checked or 0 if not checked)

3) Moderately (Q4=4 if checked or 0 if not checked)

4) Severely (Q4=5 if checked or 0 if not checked)

Q5: Have you ever experienced important punishments or dismisses in your lifetime?

1) Yes (Q5=1 if checked or 0 if not checked)

2) No (Skip to Q7)

Q6: To what extent has the experience affected you?

1) A little (Q6=2 if checked or 0 if not checked)

2) Slightly (Q6=3 if checked or 0 if not checked)

3) Moderately (Q6=4 if checked or 0 if not checked)

4) Severely (Q6=5 if checked or 0 if not checked)

Q7: Have you ever experienced important promotions or awards in your lifetime?

1) Yes (Q7=1 if checked or 0 if not checked)

2) No (Skip to Q9)

Q8: To what extent has the experience affected you?

1) A little (Q8=2 if checked or 0 if not checked)

2) Slightly (Q8=3 if checked or 0 if not checked)

3) Moderately (Q8=4 if checked or 0 if not checked)

4) Severely (Q8=5 if checked or 0 if not checked)

Q9: Have you ever experienced admirable achievements in your lifetime?

1) Yes (Q9=1 if checked or 0 if not checked)

2) No (Skip to Q11)

Q10: To what extent has the experience affected you?

1) A little (Q10=2 if checked or 0 if not checked)

2) Slightly (Q10=3 if checked or 0 if not checked)

3) Moderately (Q10=4 if checked or 0 if not checked)

4) Severely (Q10=5 if checked or 0 if not checked)

Q11: Have you ever experienced forced or disliked endeavors in your lifetime?

1) Yes (Q11=1 if checked or 0 if not checked)

2) No (Skip to Q13)

Q12: To what extent has the experience affected you?

1) A little (Q12=2 if checked or 0 if not checked)

2) Slightly (Q12=3 if checked or 0 if not checked)

3) Moderately (Q12=4 if checked or 0 if not checked)

4) Severely (Q12=5 if checked or 0 if not checked)

Q13: Have you ever experienced major accidents or mistakes in your lifetime?

1) Yes (Q13=1 if checked or 0 if not checked)

2) No (Skip to Q15)

Q14: To what extent has the experience affected you?

1) A little (Q14=2 if checked or 0 if not checked)

2) Slightly (Q14=3 if checked or 0 if not checked)

3) Moderately (Q14=4 if checked or 0 if not checked)

4) Severely (Q14=5 if checked or 0 if not checked)

Q15: Have you ever experienced natural disasters in your lifetime?

1) Yes (Q15=1 if checked or 0 if not checked)

2) No (Skip to Q17)

Q16: To what extent has the experience affected you?

1) A little (Q16=2 if checked or 0 if not checked)

2) Slightly (Q16=3 if checked or 0 if not checked)

3) Moderately (Q16=4 if checked or 0 if not checked)

4) Severely (Q16=5 if checked or 0 if not checked)

Q17: Have you ever experienced important misunderstandings or blames in your lifetime?

1) Yes (Q17=1 if checked or 0 if not checked)

2) No (Skip to Q19)

Q18: To what extent has the experience affected you?

1) A little (Q18=2 if checked or 0 if not checked)

2) Slightly (Q18=3 if checked or 0 if not checked)

3) Moderately (Q18=4 if checked or 0 if not checked)

4) Severely (Q18=5 if checked or 0 if not checked)

Q19: Have you ever experienced law suits due to yourself in your lifetime?

1) Yes (Q19=1 if checked or 0 if not checked)

2) No (Skip to Q21)

Q20: To what extent has the experience affected you?

1) A little (Q20=2 if checked or 0 if not checked)

2) Slightly (Q20=3 if checked or 0 if not checked)

3) Moderately (Q20=4 if checked or 0 if not checked)

4) Severely (Q20=5 if checked or 0 if not checked)

Q21: Have you ever experienced law suits due to your relatives in your lifetime?

1) Yes (Q21=1 if checked or 0 if not checked)

2) No (Skip to Q23)

Q22: To what extent has the experience affected you?

1) A little (Q22=2 if checked or 0 if not checked)

2) Slightly (Q22=3 if checked or 0 if not checked)

3) Moderately (Q22=4 if checked or 0 if not checked)

4) Severely (Q22=5 if checked or 0 if not checked)

Q23: Have you ever experienced long-term enmities with others in your lifetime?

1) Yes (Q23=1 if checked or 0 if not checked)

2) No (Skip to Q25)

Q24: To what extent has the experience affected you?

1) A little (Q24=2 if checked or 0 if not checked)

2) Slightly (Q24=3 if checked or 0 if not checked)

3) Moderately (Q24=4 if checked or 0 if not checked)

4) Severely (Q24=5 if checked or 0 if not checked)

Q25: Have you ever experienced marital or love breakups or conflicts in your lifetime?

1) Yes (Q25=1 if checked or 0 if not checked)

2) No (Skip to Q27)

Q26: To what extent has the experience affected you?

1) A little (Q26=2 if checked or 0 if not checked)

2) Slightly (Q26=3 if checked or 0 if not checked)

3) Moderately (Q26=4 if checked or 0 if not checked)

4) Severely (Q26=5 if checked or 0 if not checked)

Q27: Have you ever experienced major injuries or diseases of your relatives in your lifetime?

1) Yes (Q27=1 if checked or 0 if not checked)

2) No (Skip to Q29)

Q28: To what extent has the experience affected you?

1) A little (Q28=2 if checked or 0 if not checked)

2) Slightly (Q28=3 if checked or 0 if not checked)

3) Moderately (Q28=4 if checked or 0 if not checked)

4) Severely (Q28=5 if checked or 0 if not checked)

Q29: Have you ever experienced loss of relatives like parent, spouse and children?

1) Yes (Q29=1 if checked or 0 if not checked)

2) No (Skip to Q31)

Q30: To what extent has the experience affected you?

1) A little (Q30=2 if checked or 0 if not checked)

2) Slightly (Q30=3 if checked or 0 if not checked)

3) Moderately (Q30=4 if checked or 0 if not checked)

4) Severely (Q30=5 if checked or 0 if not checked)

Q31: Have you ever experienced frequent parental conflicts in your lifetime?

1) Yes (Q31=1 if checked or 0 if not checked)

2) No (Skip to Q33)

Q32: To what extent has the experience affected you?

1) A little (Q32=2 if checked or 0 if not checked)

2) Slightly (Q32=3 if checked or 0 if not checked)

3) Moderately (Q32=4 if checked or 0 if not checked)

4) Severely (Q32=5 if checked or 0 if not checked)

Q33: Do you agree you have lived a life prevailed by stressful tasks?

1) Yes (Q33=1 if checked or 0 if not checked)

2) No

Q34: To what extent can your life be described as stressful tasks prevailed?

1) A little (Q34=2 if checked or 0 if not checked)

2) Slightly (Q34=3 if checked or 0 if not checked)

3) Moderately (Q34=4 if checked or 0 if not checked)

4) Severely (Q34=5 if checked or 0 if not checked)

Q35: Do you agree that you have worried a lot about your children most of your lifetime?

1) Yes (Q35=1 if checked or 0 if not checked)

2) No

Q36: To what extent can your life be described as over worried about your children?

1) A little (Q36=2 if checked or 0 if not checked)

2) Slightly (Q36=3 if checked or 0 if not checked)

3) Moderately (Q36=4 if checked or 0 if not checked)

4) Severely (Q36=5 if checked or 0 if not checked)

Q37: Have you ever experienced financial hardship in your lifetime?

1) Yes (Q37=1 if checked or 0 if not checked)

2) No (Skip to Q39)

Q38: To what extent has the experience affected you?

1) A little (Q38=2 if checked or 0 if not checked)

2) Slightly (Q38=3 if checked or 0 if not checked)

3) Moderately (Q38=4 if checked or 0 if not checked)

4) Severely (Q38=5 if checked or 0 if not checked)

Q39: Have you ever experienced other mis-happenings in your lifetime?

1) Yes (Q39=1 if checked or 0 if not checked)

2) No

Q40: To what extent has the experience affected you?

1) A little (Q40=2 if checked or 0 if not checked)

2) Slightly (Q40=3 if checked or 0 if not checked)

3) Moderately (Q40=4 if checked or 0 if not checked)

4) Severely (Q40=5 if checked or 0 if not checked)

The Likert-scale-sum
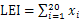


The regression-coefficients-weighted
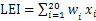


Here, i= i^th^ item of the life events included in the LE instrument, x_i_ =the Likert scale rating of the i^th^ item, W_i_=the weight of the i^th^ item generated from a logistic regression model using “any CPC” as the dependent variable (valued as “0” if no chronic physical conditions were reported and “1” if at least one CPC were reported) and x_i_ as the covariates.
