# Supplementary material for: Life events and chronic physical conditions among left-behind farmers in rural China a cross-sectional study
Source: BMC Public Health. 2015 Jul 1;15:594. doi: 10.1186/s12889-015-1877-0 (PMC4487061; doi:10.1186/s12889-015-1877-0)
Supplement: Additional file 2: — Correlation coefficients between each of the 20 life event items. [file 12889_2015_1877_MOESM2_ESM.doc]

**Additional file 3: Multivariate logistic regression statistics of the relationships between life-event index and common chronic physical conditions among farmers aged 40-55 and 56-70**

| **Farmer**  **groups** | **Chronic gastritis** | | **Prostatitis** | | **Cervicitis/** | | **Cardio/cerebro-** | | **Hypertension** | | **Diabetes** | | **Pre-diabetes** | | **Other CPCs** | | **Free from CPCs** | |
| --- | --- | --- | --- | --- | --- | --- | --- | --- | --- | --- | --- | --- | --- | --- | --- | --- | --- | --- |
|  |  |  |  |  | **vaginitis** | | **vascular dis.** | |  |  |  |  |  |  |  |  |  |  |
|  | **B** | **EXP(B)** | **B** | **EXP(B)** | **B** | **EXP(B)** | **B** | **EXP(B)** | **B** | **EXP(B)** | **B** | **EXP(B)** | **B** | **EXP(B)** | **B** | **EXP(B)** | **B** | **EXP(B)** |
| **Farmers aged 40-55** | | | | | | | | | | | | | | | | | | |
| LEI-1 | NA | NA | -1.458 | 0.233 | NA | NA | NA | NA | NA | NA | NA | NA | NA | NA | -20.066 | 0.000 | NA | NA |
| LEI-2 | 0.821 | 2.272 | -18.220 | 0.000 | 0.417 | 1.517 | 0.857 | 2.356 | 0.225 | 1.253 | -0.996 | 0.369 | -0.267 | 0.766 | -3.093 | 0.045^††^ | -0.360 | 0.698 |
| LEI-3 | 1.096 | 2.994^†^ | -1.181 | 0.307 | 1.393 | 4.028^††^ | 0.954 | 2.595 | 0.247 | 1.281 | 0.221 | 1.247 | 0.078 | 1.081 | -1.498 | 0.224^††^ | -0.596 | 0.551^†^ |
| LEI-4 | 1.176 | 3.240^†^ | -18.190 | 0.000 | 0.790 | 2.203 | 0.818 | 2.266 | 0.172 | 1.188 | 0.075 | 1.078 | 0.743 | 2.102^†^ | -1.691 | 0.184^††^ | -0.412 | 0.662 |
| LEI-5 | 1.211 | 3.358^††^ | -0.510 | 0.601 | 1.550 | 4.710^††^ | 1.043 | 2.839 | 0.213 | 1.237 | 0.305 | 1.357 | 0.662 | 1.938^†^ | -1.854 | 0.157^††^ | -0.626 | 0.535^††^ |
| LEI-6 | 1.451 | 4.267^††^ | -0.298 | 0.742 | 1.301 | 3.674^††^ | 0.929 | 2.532 | -0.013 | 0.987 | 0.025 | 1.025 | 0.440 | 1.553 | -1.073 | 0.342^††^ | -0.610 | 0.543^†^ |
| LEI-7 | 1.901 | 6.692^††^ | -1.827 | 0.161 | 1.716 | 5.561^††^ | 1.482 | 4.403 | 0.355 | 1.426 | -0.416 | 0.660 | 0.549 | 1.732 | -0.749 | 0.473 | -1.413 | 0.243^††^ |
| LEI-8 | 2.181 | 8.855^††^ | -1.844 | 0.158 | 1.160 | 3.189^†^ | 1.171 | 3.224 | 0.043 | 1.044 | 0.243 | 1.275 | 0.663 | 1.941^†^ | -0.714 | 0.489 | -0.736 | 0.479^††^ |
| LEI-9 | 2.065 | 7.883^††^ | -0.619 | 0.539 | 1.773 | 5.886^††^ | 1.394 | 4.031 | 0.318 | 1.374 | 0.311 | 1.365 | 0.820 | 2.271^††^ | -0.948 | 0.387^†^ | -0.960 | 0.383^††^ |
| LEI-10 | 1.744 | 5.720^††^ | -0.653 | 0.520 | 1.613 | 5.019^††^ | 1.776 | 5.907 | 0.542 | 1.720^†^ | -0.049 | 0.952 | 0.882 | 2.416^††^ | -0.892 | 0.410^†^ | -1.018 | 0.361^††^ |
| LEI-11 | 2.116 | 8.298^††^ | -1.068 | 0.344 | 2.263 | 9.612^††^ | 0.696 | 2.006 | 0.324 | 1.383 | 0.553 | **1.739** | 1.298 | 3.662^††^ | -0.428 | 0.652 | -1.220 | 0.295^††^ |
| LEI-12 | 1.750 | 5.755^††^ | -0.620 | 0.538 | 1.990 | 7.316^††^ | 1.844 | **6.322**^††^ | 0.396 | 1.486 | -0.105 | 0.900 | 1.252 | 3.497^††^ | -0.663 | 0.515 | -1.315 | 0.269^††^ |
| LEI-13 | 2.072 | 7.942^††^ | -0.560 | 0.571 | 2.200 | 9.021^††^ | 0.938 | 2.554 | 0.254 | 1.289 | 0.483 | 1.621 | 0.952 | 2.592^††^ | -0.049 | 0.952 | -1.394 | 0.248^††^ |
| LEI-14 | 2.450 | **11.592**^††^ | -0.802 | 0.448 | 2.444 | **11.515**^††^ | 1.783 | 5.947^††^ | 0.555 | **1.742** | 0.240 | 1.271 | 1.029 | 2.797^††^ | -0.334 | 0.716 | -1.643 | 0.193^††^ |
| LEI-15 | 2.325 | 10.226^††^ | NA | NA | 2.262 | 9.599^††^ | 1.463 | 4.321 | 0.408 | 1.504 | 0.386 | 1.471 | 1.200 | **3.319**^††^ | NA | NA | -1.685 | **0.185**^††^ |
| Constant | -3.148 | 0.043 | -5.285 | 0.005 | -0.649 | 0.523 | -6.364 | 0.002 | -4.637 | 0.010 | -1.506 | 0.222 | -0.379 | 0.685 | -0.713 | 0.490 | 1.576 | 4.836 |
| **Farmers aged 56-70 years** | | | | | | | | | | | | | | | | | | |
| LEI-1 | NA | NA | -18.000 | 0.000 | NA | NA | NA | NA | NA | NA | NA | NA | NA | NA | -2.996 | 0.050 | NA | NA |
| LEI-2 | -0.192 | 0.825 | -1.679 | 0.187 | -1.108 | 0.330 | -0.055 | 0.946 | 0.123 | 1.131 | 0.702 | 2.017 | 0.328 | 1.388 | -2.240 | 0.106 | -0.333 | 0.717 |
| LEI-3 | 0.248 | 1.282 | -0.472 | 0.624 | 0.145 | 1.156 | -0.067 | 0.935 | -0.081 | 0.922 | 0.590 | 1.804 | 0.835 | 2.304^†^ | -1.310 | 0.270 | -0.086 | 0.918 |
| LEI-4 | 0.324 | 1.383 | -0.239 | 0.787 | -1.145 | 0.318 | 0.033 | 1.033 | -0.205 | 0.814 | 0.502 | 1.651 | 0.747 | 2.110 | -1.357 | 0.257 | -0.308 | 0.735 |
| LEI-5 | 0.898 | 2.456 | -0.930 | 0.394 | 0.860 | 2.363 | 0.526 | 1.692 | 0.005 | 1.005 | 0.275 | 1.317 | 0.632 | 1.881 | -1.087 | 0.337 | -0.673 | 0.510^†^ |
| LEI-6 | 0.733 | 2.081 | -0.341 | 0.711 | -0.166 | 0.847 | 0.240 | 1.271 | -0.055 | 0.946 | 0.379 | 1.460 | 0.911 | 2.486^†^ | -0.684 | 0.505^†^ | -0.592 | 0.553 |
| LEI-7 | 0.974 | 2.649^†^ | 0.170 | 1.186 | 0.823 | 2.278 | 0.260 | 1.297 | 0.330 | 1.390 | 0.389 | 1.476 | 0.762 | 2.143 | -1.599 | 0.202 | -0.934 | 0.393^††^ |
| LEI-8 | 1.175 | 3.240^††^ | 0.614 | 1.848 | 1.315 | 3.725 | 0.251 | 1.285 | 0.250 | 1.284 | -0.096 | 0.909 | 0.981 | 2.666^†^ | -1.268 | 0.281 | -1.069 | 0.344^††^ |
| LEI-9 | 1.319 | 3.741^††^ | 0.178 | 1.194 | 1.366 | 3.919 | 0.682 | 1.977 | -0.152 | 0.859 | 0.771 | 2.162 | 0.990 | 2.692^††^ | -0.629 | 0.533^†^ | -0.937 | 0.392^††^ |
| LEI-10 | 1.001 | 2.722^†^ | 0.094 | 1.098 | 1.371 | 3.937 | 0.305 | 1.356 | 0.173 | 1.189 | 0.197 | 1.218 | 1.096 | 2.993^††^ | -1.096 | 0.334 | -0.735 | 0.479^†^ |
| LEI-11 | 1.280 | 3.598^††^ | 0.237 | 1.267 | 1.415 | 4.117 | 0.685 | **1.984** | 0.254 | 1.289 | 0.002 | 1.002 | 0.795 | 2.215^†^ | -0.300 | 0.741^††^ | -0.932 | 0.394^††^ |
| LEI-12 | 1.529 | 4.612^††^ | 0.439 | 1.552 | 1.323 | 3.756 | 0.654 | 1.923 | 0.373 | 1.452 | 0.831 | **2.296** | 1.291 | 3.638^††^ | -0.151 | 0.860^††^ | -1.177 | 0.308^††^ |
| LEI-13 | 1.702 | **5.486**^††^ | -0.198 | 0.820 | 1.256 | 3.512 | 0.640 | 1.897 | 0.180 | 1.197 | 0.626 | 1.871 | 1.356 | **3.881**^††^ | -0.361 | 0.697 | -1.480 | 0.228^††^ |
| LEI-14 | 1.384 | 3.993^††^ | 0.372 | 1.450 | 1.677 | 5.350 | 0.375 | 1.455 | 0.381 | **1.463** | 0.676 | 1.967 | 1.218 | 3.381^††^ | -0.143 | 0.867^††^ | -1.298 | 0.273^††^ |
| LEI-15 | 1.630 | 5.106^††^ | NA | NA | 1.702 | **5.484** | 0.638 | 1.892 | 0.183 | 1.201 | 0.263 | 1.301 | 1.289 | 3.628^††^ | NA | NA | -1.545 | **0.213**^††^ |
| Constant | -2.020 | 0.133 | -9.947 | 0.000 | -0.615 | 0.541 | -5.980 | 0.003 | -2.834 | 0.059 | -4.347 | 0.013 | -1.753 | 0.173 | 1.601 | 4.960 | 3.326 | 27.839 |

Note: 1) “†” and “††” denotes p<0.05 and p<0.01 respectively for the power test of null difference between age groups; 2) LEI stands for combined life event index and LEI-1 through to LEI-15, first through to fifteenth 15 percentile of LEI respectively; 3) hypertension denotes systolic/diastolic blood pressure ≥ 140/90mmHg; 4) diabetes denotes fasting capillary glucose ≥7.0mmol/L and pre-diabetes denotes fasting capillary glucose = [6.1, 6.9] mmol/L; 5) the statistics for prostatitis and other CPCs were calculated using LEI-15 (instead of LEI-1) as the reference group since there were no cases among the LEI-1 group.
