# Supplementary material for: Life events and chronic physical conditions among left-behind farmers in rural China a cross-sectional study
Source: BMC Public Health. 2015 Jul 1;15:594. doi: 10.1186/s12889-015-1877-0 (PMC4487061; doi:10.1186/s12889-015-1877-0)
Supplement: Additional file 3: — Age stratified odd ratios of chronic physical conditions between left behind farmers with different life event index. [file 12889_2015_1877_MOESM3_ESM.docx]

| **Additional file 2: Correlation coefficients between each of the 20 life event items.** | | | | | | | | | | | | | | | | | | | | |
| --- | --- | --- | --- | --- | --- | --- | --- | --- | --- | --- | --- | --- | --- | --- | --- | --- | --- | --- | --- | --- |
|  | X1 | X2 | X3 | X4 | X5 | X6 | X7 | X8 | X9 | X10 | X11 | X12 | X13 | X14 | X15 | X16 | X17 | X18 | X19 | X20 |
| X1 |  |  |  |  |  | X1: Schooling/examination failures; X2: Abandonment of favorite pursues; X3: Punishments/dismisses; X4: Promotions/awards;X5: Admirable achievements; X6: Forced/ disliked endeavors; X7: Accidents/mistakes; X8: Natural disasters; X9: Misunderstandings/ blames; X10: Law suits due to self; X11: Law suits due to relatives; X12: Enmities with others; X13: Marital/love breakups/conflicts; X14: Major injuries/diseases of relatives;X15: Loss of relatives; X16: Frequent parental conflicts; X17: Over worries about children; X18: Financial hardship;X19: Stressful tasks prevailed life; X20: Other mis-happenings;   \|  \| \| --- \| |  |  |  |  |  |  |  |  |  |  |  |  |  |  |
| X2 | .162^**^ |  |  |  |  |  |  |  |  |  |  |  |  |  |  |  |  |  |  |  |
| X3 | .088^**^ | .144^**^ |  |  |  |  |  |  |  |  |  |  |  |  |  |  |  |  |  |  |
| X4 | .061^**^ | .083^**^ | .030 |  |  |  |  |  |  |  |  |  |  |  |  |  |  |  |  |  |
| X5 | .167^**^ | .203^**^ | .094^**^ | .229^**^ |  |  |  |  |  |  |  |  |  |  |  |  |  |  |  |  |
| X6 | .088^**^ | .246^**^ | .133^**^ | .098^**^ | .239^**^ |  |  |  |  |  |  |  |  |  |  |  |  |  |  |  |
| X7 | .015 | .092^**^ | .081^**^ | .034^*^ | .090^**^ | .107^**^ |  |  |  |  |  |  |  |  |  |  |  |  |  |  |
| X8 | .061^**^ | .108^**^ | .122^**^ | .070^**^ | .085^**^ | .075^**^ | .051^**^ |  |  |  |  |  |  |  |  |  |  |  |  |  |
| X9 | .158^**^ | .165^**^ | .162^**^ | .061^**^ | .173^**^ | .205^**^ | .122^**^ | .119^**^ |  |  |  |  |  |  |  |  |  |  |  |  |
| X10 | .003 | .050^**^ | .083^**^ | .042^**^ | .024 | .059^**^ | .138^**^ | .031^*^ | .121^**^ |  |  |  |  |  |  |  |  |  |  |  |
| X11 | .012 | .028 | .053^**^ | .031^*^ | .027 | .040^*^ | .048^**^ | .007 | .047^**^ | .078^**^ |  |  |  |  |  |  |  |  |  |  |
| X12 | .080^**^ | .116^**^ | .119^**^ | .045^**^ | .101^**^ | .153^**^ | .124^**^ | .109^**^ | .259^**^ | .108^**^ | .084^**^ |  |  |  |  |  |  |  |  |  |
| X13 | .079^**^ | .091^**^ | .023 | .038^*^ | .086^**^ | .070^**^ | .051^**^ | .035^*^ | .107^**^ | .048^**^ | .019 | .068^**^ |  |  |  |  |  |  |  |  |
| X14 | .026 | .030 | .077^**^ | .024 | .055^**^ | .039^*^ | .068^**^ | .088^**^ | .094^**^ | .049^**^ | .079^**^ | .057^**^ | -.003 |  |  |  |  |  |  |  |
| X15 | .053^**^ | .045^**^ | .024 | .050^**^ | .051^**^ | .042^**^ | .047^**^ | .095^**^ | .040^*^ | -.004 | .001 | .062^**^ | -.016 | .064^**^ |  |  |  |  |  |  |
| X16 | .077^**^ | .057^**^ | .077^**^ | .019 | .047^**^ | .089^**^ | .074^**^ | .050^**^ | .134^**^ | .047^**^ | .068^**^ | .084^**^ | .051^**^ | .052^**^ | .021 |  |  |  |  |  |
| X17 | .168^**^ | .051^**^ | .065^**^ | .004 | .048^**^ | .034^*^ | .021 | .100^**^ | .100^**^ | -.019 | .017 | .056^**^ | -.020 | .059^**^ | .112^**^ | .039^*^ |  |  |  |  |
| X18 | .119^**^ | .070^**^ | .039^*^ | .001 | .015 | .021 | .021 | .072^**^ | .087^**^ | -.006 | .001 | .059^**^ | .017 | .080^**^ | .135^**^ | -.003 | .291^**^ |  |  |  |
| X19 | .141^**^ | .169^**^ | .123^**^ | -.037^*^ | .101^**^ | .148^**^ | .063^**^ | .080^**^ | .155^**^ | .022 | .040^*^ | .155^**^ | .067^**^ | .096^**^ | .077^**^ | .062^**^ | .177^**^ | .206^**^ |  |  |
| X20 | .068^**^ | .056^**^ | .048^**^ | .024 | .063^**^ | .052^**^ | .055^**^ | .037^*^ | .103^**^ | .030 | .029 | .125^**^ | .054^**^ | .033^*^ | .017 | .052^**^ | .045^**^ | .031^*^ | .072^**^ |  |
| “*” and “**” denote p<0.05 and p<0.01 respectively for the power test of null difference between LE items. | | | | | | | | | | | | | | | | | | | | |
